# Optimization of sniff nasal inspiratory pressure (SNIP) measurement methodology in healthy subjects

**DOI:** 10.1186/s12890-023-02348-0

**Published:** 2023-02-15

**Authors:** R. J. Wilding, M. Thynne, M. M. F. Subhan

**Affiliations:** 1 University Hospitals Plymouth NHS Trust, Plymouth, PL6 8DH UK; 2 Chest Clinic, University Hospitals Plymouth NHS Trust, Plymouth, PL6 8DH UK; 3 School of Biomedical Sciences, Faculty of Health, University of Plymouth, C507, Portland Square, Drake Circus, Plymouth, PL4 8AA UK

**Keywords:** SNIP, Respiratory muscle strength, Reproducibility, Healthy subjects

## Abstract

**Background:**

Maximal inspiratory pressure (MIP) is currently the most commonly used measure for respiratory muscle strength (RMS) estimation, however, requires significant effort. Falsely low values are therefore common, especially in fatigue-prone subjects, such as neuromuscular disorder patients. In contrast, sniff nasal inspiratory pressure (SNIP) requires a short, sharp sniff; this is a natural manoeuvre, decreasing required effort. Consequently, it has been suggested that use of SNIP could confirm the accuracy of MIP measurements. However, no recent guidelines regarding the optimal method of SNIP measurement exist, and varied approaches have been described.

**Objectives:**

We compared SNIP values from three conditions, namely with 30, 60 or 90 s time intervals between repeats, the right (SNIP_R_) and left (SNIP_L_) nostril, and the contralateral nostril occluded (SNIP_O_) or non-occluded (SNIP_NO_). Additionally, we determined the optimal number of repeats for accurate SNIP measurement.

**Method:**

52 healthy subjects (23 males) were recruited for this study, of which a subset of 10 subjects (5 males) completed tests comparing the time interval between repeats. SNIP was measured from functional residual capacity via a probe in one nostril, while MIP was measured from residual volume.

**Results:**

There was no significant difference in SNIP depending on the interval between repeats (*P* = 0.98); subjects preferred the 30 s. SNIP_O_ was significantly higher than SNIP_NO_ (*P* < 0.00001) but SNIP_L_ and SNIP_R_ did not significantly differ (*P* =  0.60). There was an initial learning effect for the first SNIP test; SNIP did not decline during 80 repeats (*P* =  0.64).

**Conclusions:**

We conclude that SNIP_O_ is a more reliable RMS indicator than SNIP_NO_, as there is reduced risk of RMS underestimation. Allowing subjects to choose which nostril to use is appropriate, as this did not significantly affect SNIP, but may increase ease of performance. We suggest that twenty repeats is sufficient to overcome any learning effect and that fatigue is unlikely after this number of repeats. We believe these results are important in aiding the accurate collection of SNIP reference value data in the healthy population.

**Supplementary Information:**

The online version contains supplementary material available at 10.1186/s12890-023-02348-0.

## Introduction

Estimation of respiratory muscle strength (RMS) by measuring pressures within the thoracic cavity aids diagnosis and monitoring of several conditions, such as neuromuscular disorders (NMDs; [1]). Such techniques are beneficial, as pressure-based values typically decline before volume-based measures such as vital capacity [1], allowing more timely diagnosis and treatment initiation, which can improve prognosis and quality of life. Maximal inspiratory pressure (MIP) is currently the most commonly used RMS measure [2], however it demands significant effort, as it requires a maximum inspiration from residual volume [3]. This is disadvantageous to NMD patients, as they are prone to fatigue, meaning RMS underestimation is common when measuring MIP in these patients [4]. MIP performance also requires a tight seal to be formed between the mouthpiece and the lips; facial muscle weakness is common in NMDs, and may impair adequate seal formation, again conferring risk of RMS underestimation [4]. This can result in overdiagnosis and inappropriate treatment, impairing the quality of life of patients.

In contrast to MIP, sniff nasal inspiratory pressure (SNIP) measurement does not require formation of such a seal, and requires relatively little effort [5]. SNIP is a non-invasive measure of RMS, measured via a probe inserted into one nostril during a short, sharp sniff [6]. The sniff is dependent on inspiratory muscle contraction, predominately the diaphragm, therefore the pressures recorded indicate RMS [7]. SNIP measurements have been correlated with quality of life and mortality risk in conditions including amyotrophic lateral sclerosis and are an indication for therapy initiation [1, 8]. Several groups have compared SNIP and MIP; one has shown SNIP to be higher [9] while others have shown no difference [10, 11]. It has been suggested that SNIP is unlikely to replace MIP, however several groups have concluded that use of both tests in a complementary manner could be beneficial [9, 12, 13]. For example, recording a low MIP yet a normal SNIP could indicate issues such as suboptimal effort, fatigue or lack of a complete seal during MIP performance, helping to rule out inspiratory muscle weakness and minimizing false positives. This would mean clinicians gain a more accurate idea of patients’ RMS, increasing diagnostic and prognostic accuracy and ensuring the most appropriate therapy is administered [14].

Accurate SNIP measurement is vital due to the importance of the values in diagnosing and monitoring disease, both alone and alongside measures such as MIP. However, varied measurement methodologies have been described. Initial SNIP methodology stated the contralateral nostril should be non-occluded [6], however, several groups have subsequently detailed methodology with occlusion of this nostril [2, 10, 15–17]. Additionally, no consensus exists regarding which nostril should be used; often the nostril appearing most patent is chosen [10, 17] or where a few SNIP tests show a higher value [18], however the impact of nostril choice on SNIP values has received limited attention. The optimal number of repeats has also been a subject of debate. Lofaso et al. reported 10 repeats is sufficient to complete the learning effect [19], however other groups have detailed methodology using both more [4, 20, 21] and less repeats [8, 16, 22]. In addition to repeat number, the optimal rest time between repeats is poorly defined. The impact of this variable is important to consider, as too short an interval could increase risk of fatigue, whereas too long an interval could pose risk of the subject becoming distracted and consequently applying submaximal effort. The present study will determine the impact of the above variables on SNIP values, thus clarifying these discrepancies and increasing SNIP reliability. Better standardizations would enable the collection of accurate reference values in healthy subjects, which will allow better assessment of patient populations.

Our aims included to test the impact of the time interval between SNIP repeats, compare right and left nostril data and determine whether occluding the contralateral nostril influenced results. Additionally, we investigated if there was a learning effect and whether subjects were likely to experience fatigue.

## Methods

The study was approved by the Faculty of Science and Engineering Research Ethics Committee, University of Plymouth. The subjects were recruited randomly from staff and students of the University of Plymouth. All subjects gave informed and written consent before participation. The experimental protocol was explained to all subjects.

### Participants

52 healthy subjects (23 males) participated in the study. Exclusion criteria included being under 18 or over 65 years of age, smokers, patients with cardiorespiratory or neuromuscular disease or any previous major cardiothoracic surgery. We verified that subjects met these criteria via a questionnaire. One subject’s results were excluded from analyses due to consistent SNIP readings of zero, indicating inadequate SNIP performance.

### Measurements

Prior to SNIP performance, anthropometric and other measurements were recorded, including height (Seca, Germany), weight (Marsden, UK), blood pressure, heart rate and SpO_2_; the last three measurements were recorded using a Vital Signs Monitor 300 (Welch Allyn, USA). MIP and maximal expiratory pressure (MEP) were recorded using a MicroRPM device (Care Fusion, UK) and variability was less than 20% [3]. For MIP, subjects exhaled to residual volume before inspiring through a mouthpiece until recording a maximum value, and for MEP, subjects inhaled to total lung capacity before expiring through the device [3, 18]. Pulmonary function tests (PFTs) were performed using a Micro Loop spirometer (Care Fusion, UK), and repeated until meeting British Thoracic Society criteria [23].

**Fig. 1:**
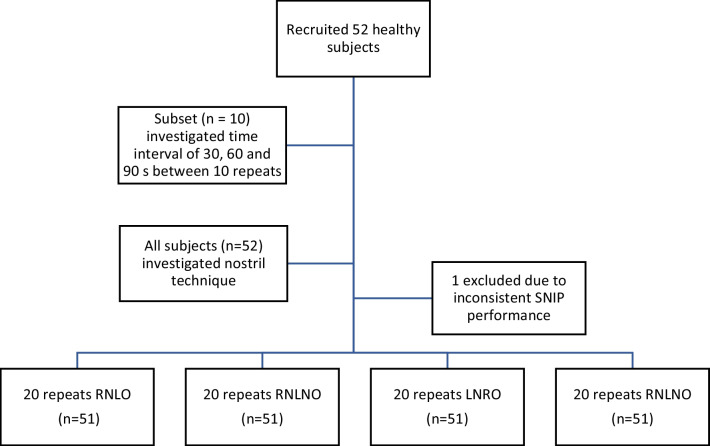
Subject recruitment and study design. RNLO: right nostril, left occluded; RNLNO: right nostril, left non-occluded; LNRO: left nostril, right occluded; LNRNO: left nostril, right non-occluded

SNIP was measured using the MicroRPM device whilst subjects remained seated upright with both feet on the floor. Instructions were given to breathe normally between tests, and, on cue, exhale to functional residual capacity before sharply sniffing inwards with the mouth closed [18]. With a subset of ten subjects (five males), SNIP was measured via a probe inserted into the right nostril, with the left non-occluded. Subjects performed three sets of ten repeats; during each, either a 30, 60 or 90 s rest was given between repeats (Fig. 1). The order of tests was randomized for each subject. Data from these experiments determined that 30 s was an appropriate rest interval for all remaining experiments. After an interval of at least one week, all 51 subjects performed four sets of 20 SNIP tests (Fig. 1). Each set used a different technique to measure SNIP; via the right nostril with the left non-occluded (RNLNO), the right nostril with the left occluded (RNLO), the left nostril with the right non-occluded (LNRNO) or the left nostril with the right occluded (LNRO). The order of tests was randomized for each subject by giving them four numbered cards, where each number corresponded to one of the four techniques, and asking the subjects to pick these in a sequence (whilst the cards were face down). The contralateral nostril was occluded by subjects placing their thumb over their nostril for the RNLO and LNRO techniques.

### Statistical analysis

For analysis purposes, the mean maximal SNIP (mmSNIP) was calculated. This was calculated by selecting each subject’s maximal SNIP for each technique and then dividing the sum of these by the number of subjects. For example, the maximal SNIP values for the subset investigating the 30 s interval, would be summed and then divided by ten (number of subset subjects). Similarly, when investigating nostril technique, mmSNIP was calculated by summing the maximum SNIP values and dividing by the total subject number (n = 51). Repeated-measures ANOVA tested differences between the mmSNIP obtained from the four techniques and also tested differences across all 20 repeats within each set. Unpaired t-testing compared differences in SNIP depending on gender, ethnicity, nostril used and occlusion. Multiple linear regression was used the individual maximal SNIP values (SNIP_max_) as the dependent variable and with gender, age, BMI, physical activity, mean arterial pressure, heart rate, SpO2 and lung function as independent variables in the model. All statistical analyses were performed using SPSS Version 24, with a probability level *P* < 0.05 considered statistically significant for t-tests and multiple linear regression. When comparing the four techniques, a stricter probability level of *P* < 0.0125 was used for the repeated-measures ANOVA, with Bonferroni’s correction being used to adjust for type 1 errors. However, with the 10 or 20 repeat experiments, a probability level *P* < 0.05 was used [24]. Bland–Altman analysis looks for agreement between two methods of measurement. The maximum SNIP data minus maximum MIP was run through a one group t-test, with a population mean of 0. If the values were significantly different from the mean of 0, then it was assumed that the two values differed from each other. Subject data can be viewed in this Additional file 1: Supplementary data file 1.

## Results

### Participants

Mean questionnaire and lung function data of the 51 study participants are summarised in Table 1. No subject experienced any adverse events whilst participating in the study. Each mean SNIP value in this table was calculated using 2,040 individual SNIP measurements, 51 subjects had 20 tests taken twice. The mean value of the maximal SNIP for each of these 20 tests (102 values) was used.

**Table 1:** Mean (± SD) data of the 51 participants

Variables	Mean ± SD
Age (years)	25.5 ± 14.5
BMI (kg/m^2^)	26.1 ± 6.4
SpO_2_ (%)	98.5 ± 1.0
Heart rate (bpm)	76.2 ± 13.7
SBP (mmHg)	117.4 ± 13.1
DBP (mmHg)	71.1 ± 11.6
Alcohol (units/week)	2.3 ± 3.4
Physical activity (sessions/week)	2.9 ± 2.1
FVC (% predicted)	94.8 ± 12.9
FEV_1_ (% predicted)	93.5 ± 15.6
FEV_1_/FVC (% predicted)	102.2 ± 12.1
PEF (% predicted)	89.9 ± 25.1
MIP (cmH_2_O)	72.8 ± 33.9
MEP (cmH_2_O)	80.9 ± 35.5
Contralateral nostril occluded SNIP (SNIP_O_; cmH_2_O)	73.4 ± 33.7
Contralateral nostril non-occluded SNIP (SNIP_NO_; cmH_2_O)	56.1 ± 27.6
Left nostril SNIP (SNIP_L_; cmH_2_O)	63.6 ± 31.1
Right nostril SNIP (SNIP_R_; cmH_2_O)	66.0 ± 32.9
Maximum SNIP value across all 80 tests (SNIP_max_; cmH_2_O)	82.0 ± 33.7

BMI: body mass index; SpO_2_: peripheral oxygen saturation; S and DBP: systolic and diastolic blood pressure; FVC: forced vital capacity; FEV_1_: forced expiratory volume in 1 s; PEF: peak expiratory flow; MIP: maximum inspiratory pressure; MEP: maximum expiratory pressure; SNIP: sniff nasal inspiratory pressure

### Effect of time interval between SNIP tests (n = 10)

The mmSNIP did not significantly differ depending on whether repeats were performed 30, 60 or 90 s apart (84.0 ± 30.9, 84.6 ± 32.6 and 86.9 ± 34.3 cmH_2_O; *P* =  0.98, n = 10). In terms of the actual order these tests were performed, there was no significant difference in the mmSNIP obtained from tests in the first, second or third (85.6 ± 35.1, 85.3 ± 30.6 and 84.6 ± 32.1 cmH_2_O) position of order (*P* =  0.95), irrespective of which repeat time interval was used.

### Impact of nostril and occlusion choice on SNIP

**Fig. 2:**
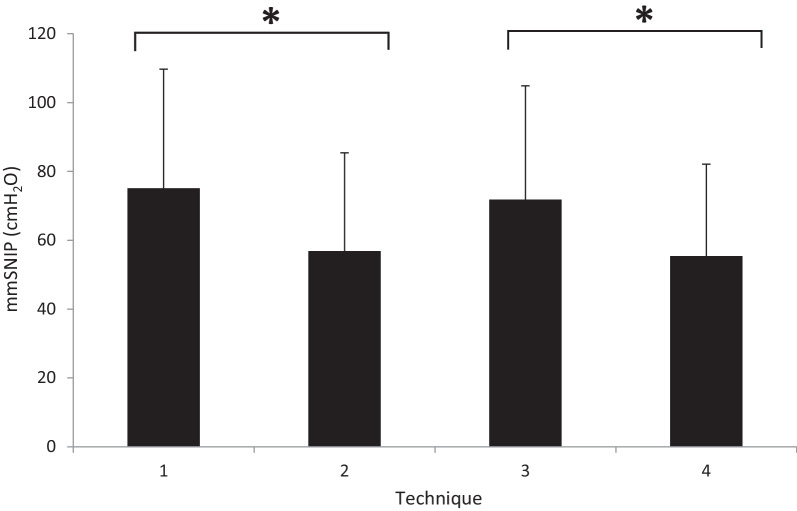
Mean maximal SNIP (± SD) obtained using different nostril techniques (n = 51). RNLO: right nostril, left occluded; RNLNO: right nostril, left non-occluded; LNRO: left nostril, right occluded; LNRNO: left nostril, right non-occluded. * denotes significant differences (*P* < 0.01) between RNLO vs. RNLNO and LNRO vs. LNRNO

Overall, mmSNIP significantly differed across the four techniques tested (*P* < 0.00001, n = 51; Fig. 2). Significant differences between tests with the contralateral nostril occluded (SNIP_O_; RNLO and LNRO) and non-occluded (SNIP_NO_; RNLNO and LNRNO) were found using t-testing (73.4 ± 33.7 vs. 56.1 ± 27.6 cmH_2_O, *P* < 0.00001, n = 102). However, when tests were performed via the left (SNIP_L_; LNRO and LNRNO) or right (SNIP_R_; RNLO and RNLNO) nostril, no difference was noted (63.6 ± 31.1 vs. 66.0 ± 32.9 cmH_2_O, *P* =  0.59, n = 102). T-testing also revealed significant differences between the RNLO and RNLNO techniques (75.1 ± 34.6 vs. 56.8 ± 28.6 cmH_2_O; *P* =  0.005, n = 51), and between the LNRO and LNRNO techniques (71.8 ± 33.1 vs. 55.4 ± 26.8 cmH_2_O; *P* =  0.007, n = 51). However, no significant difference was found between the RNLO and LNRO techniques (75.1 ± 34.6 vs. 71.8 ± 33.1 cmH_2_O; *P* =  0.54, n = 51), or between the RNLNO and LNRNO techniques (56.8 ± 28.6 vs. 55.4 ± 26.8 cmH_2_O; *P* =  0.79, n = 51).

### Presence of a learning effect or fatigue

**Fig. 3:**
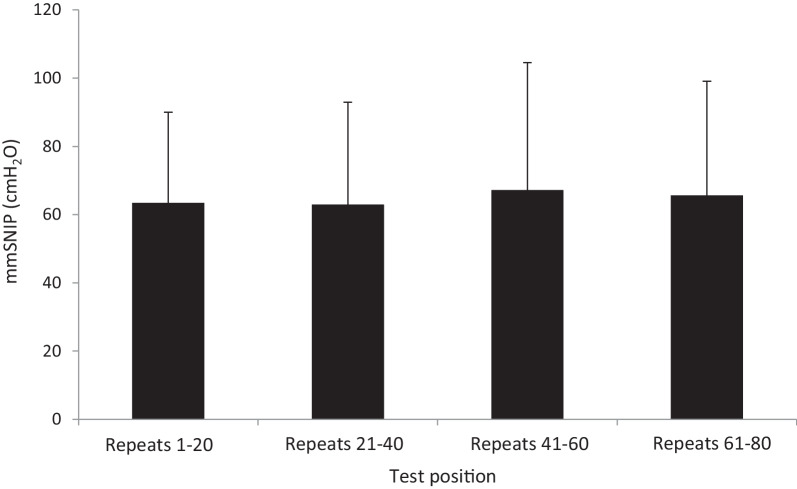
Mean maximal SNIP (± SD) obtained from each set of 20 SNIP tests (n = 51); mmSNIP did not significantly differ depending on the set position (*P* =  0.64)

Repeated-measures ANOVA compared mmSNIP data for tests in the actual order they were performed, regardless of technique used. No significant difference was found between the mmSNIP obtained in the first, second, third or fourth set of 20 repeats (63.4 ± 26.6, 62.9 ± 30.0, 67.1 ± 37.4 and 65.6 ± 33.5 cmH_2_O; *P* =  0.64; Fig. 3).

**Fig. 4:**
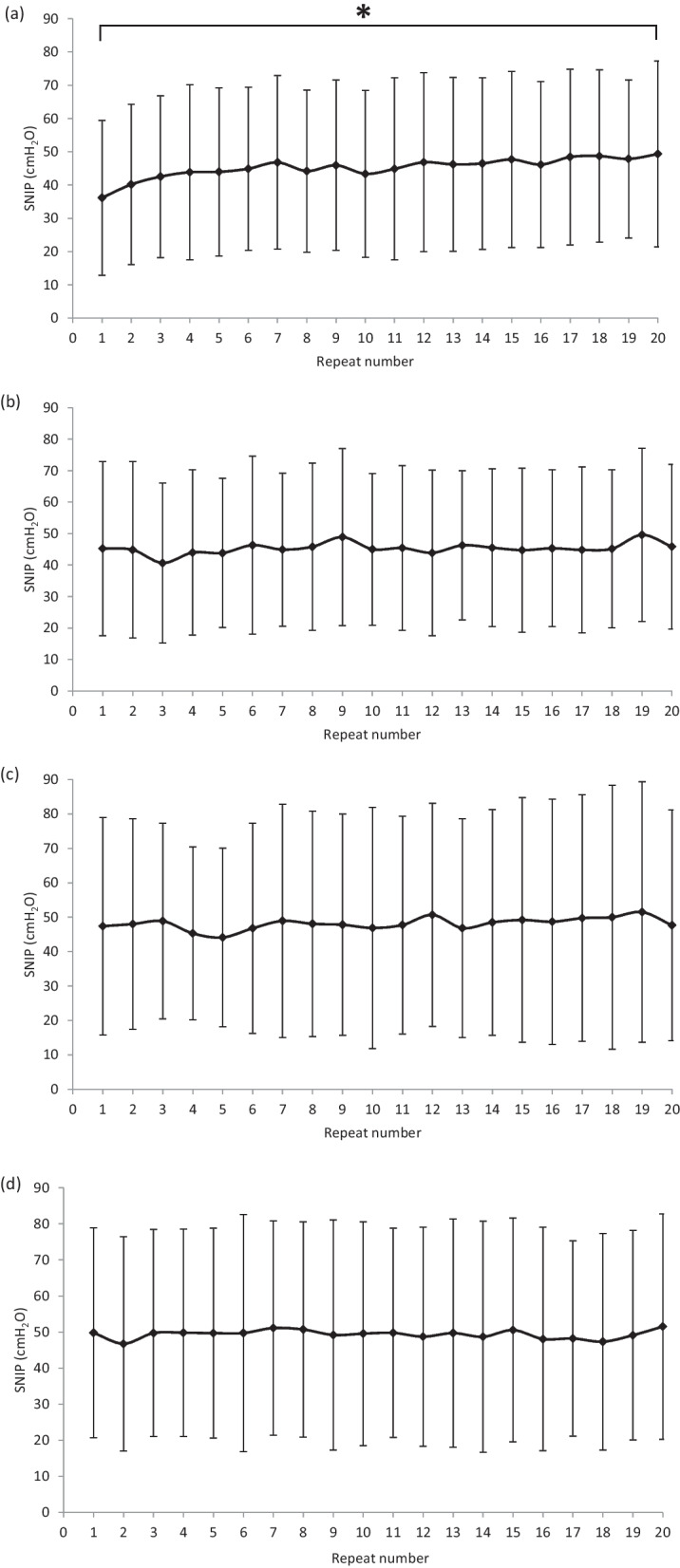
Mean (± SD) SNIP data obtained by subjects within the **a** first, **b** second, **c** third and **d** fourth sets for tests in the actual order they were performed, irrespective of technique used (n = 51). * denotes significant difference in mean SNIP between repeat number 1 and 20 (*P* =  0.006 within (a), the first set)

However, there was an overall significant difference in the mean SNIP between repeats within the first set of 20 SNIPs (*P* =  0.00006), indicating the presence of a learning effect within the first 20 repeats. Using within-subject pairwise comparisons showed a significant difference between repeat number 1 and repeat numbers 7, 9 and 12–20 (*P* < 0.05). This was not the case for repeats performed in the second (*P* =  0.21), third (*P* =  0.36) or fourth (*P* =  0.70) sets (Fig. 4).

### Relationship between SNIP and gender, ethnicity, MIP and MEP

**Fig. 5:**
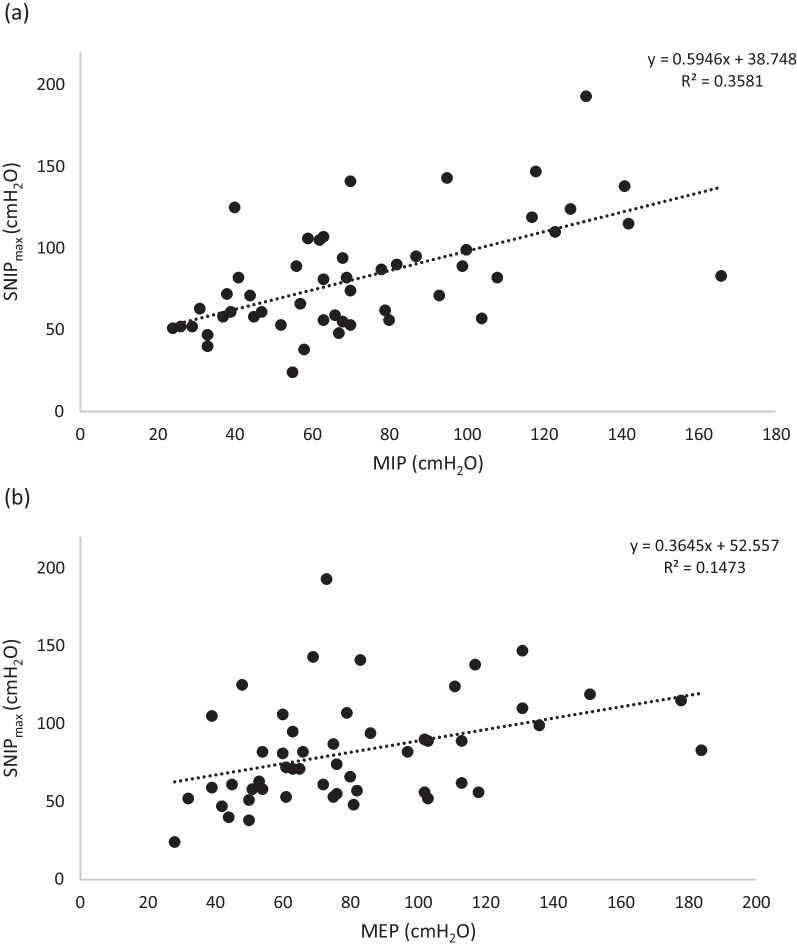
Relationship between **a** SNIP_max_ and MIP, showing a significant correlation **b** SNIP_max_ and MEP, showing no significant correlation, in our cohort of healthy subjects (n = 51)

T-tests revealed that mmSNIP did not significantly differ between males and females (87.7 ± 32.9 vs. 76.1 ± 34.4 cmH_2_O, *P* =  0.23). There was also no statistically significant difference in mmSNIP between Caucasians and non-Caucasians (80.5 ± 4.9 vs. 89.5 ± 20.8 cmH_2_O, *P* =  0.62), however, there were only four non-Caucasians in our study. Using multiple linear regression with SNIP_max_ as the dependant variable, our model found MIP to be correlated with SNIP (*P* =  0.024; Fig. 5). No other non-SNIP variables were significantly correlated with SNIP.

### Bland–Altman analysis for SNIP_max_ and MIP

**Fig. 6:**
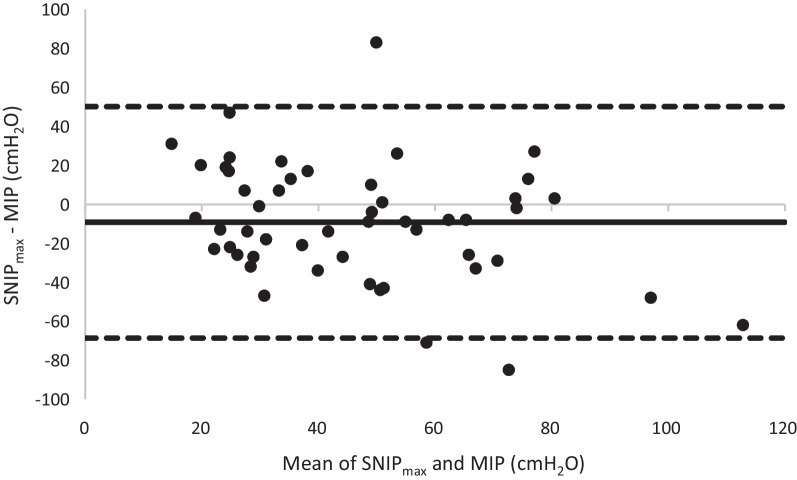
Bland–Altman showing the difference in SNIP_max_ and MIP, plotted against the mean of these two values (n = 51). The upper dashed line indicates the upper limit of agreement, the continuous line the mean difference and the lower dashed line indicates the lower limit of agreement

Although linear regression found a relationship between the maximum values of SNIP and MIP, a Bland–Altman analysis was required. From a Bland–Altman plot, we did find three data points being beyond the limits of agreement (Fig. 6). A one sample t-test showed SNIP_max_ was significantly different, and greater, than MIP (82.0 ± 33.7 vs. 72.8 ± 33.9cmH_2_O, *P* =  0.034).

## Discussion

This study has clearly shown that 30 s intervals between repeat tests are appropriate in SNIP testing in healthy subjects. We have also demonstrated that techniques occluding the contralateral nostril significantly raised SNIP values, but using either the right or left nostril did not significantly differ. There was also a learning effect, where the first test in the first set of 20 SNIP tests (out of four sets), was seen to be lower. Although recent data has been published on the effects of contralateral occlusion on SNIP values, there is a scarcity of data on intervals between tests and differences between right and left nostrils.

### Effect of interval between tests on SNIP values

Results obtained from a subset of subjects led us to conclude that the rest time between intervals does not significantly impact SNIP. The shortest rest time (30 s) was tolerable to participants; no subject complained this was too short and no adverse events occurred. Additionally, several subjects thought 90 s intervals were too long and increased the risk of distraction; this could lead to a submaximal effort. This is contrary to our hypothesis that a longer rest time would be preferable to subjects. As far as the authors are aware, we are the first group to investigate whether different time intervals affect SNIP values. We did not investigate how intervals shorter than 30 s affected SNIP or subject comfort, therefore cannot conclude whether use of shorter rest times would be appropriate. However, in our study, we turned our MicroRPM device off after each test, so we could record each SNIP value. If not turned off, the device would only record a value if it were greater than a previous reading, so a repeat lower reading would not be displayed. Although this is clinically useful, we wanted to record each individual value, thus turned the device off, meaning 5 to 10 s was needed for it to restart each time. Taking this into account and the fact the investigator needed to record the measurement, it would have been difficult to complete this process within an interval less than 30 s. As the present study included only healthy subjects, we cannot conclude whether 30 s is an appropriate rest interval for patients who may be prone to fatigue, such as those with NMDs. However, our findings are supported by the fact that 30 s intervals have previously been used in NMD and lung disease patients, with no adverse events or significant impact on SNIP values reported [11, 19] and in healthy children [25].

### Comparison of SNIP_O_ and SNIP_NO_

Our data showed a significant difference between SNIP_O_ and SNIP_NO_; mean maximum SNIP_O_ was significantly higher than mean maximum SNIP_NO_, in agreement with previous reports [10, 16, 17, 26]. However, our mmSNIP values were slightly lower than previously reported [10, 16, 26] and one possibility for this could be differences in the age of these cohorts [27]. The percentage difference between occluded and non-occluded SNIP values were similar; ours was 31% and previous groups showed it to be 29% [10] and 26% [16]. However for one study it was 8% [26].

The difference between SNIP_O_ and SNIP_NO_ is most likely because occlusion ensures a completely sealed nasal cavity rather than relying on collapse of the contralateral nasal valve, as the open manoeuvre must [28], increasing the recorded pressure. In both the present study and previous studies [10, 16, 17], SNIP_NO_ was lower than MIP. SNIP_NO_ may therefore be more likely to underestimate RMS, therefore may not be a suitable for use after a low MIP has been recorded. In contrast, we found SNIP_O_ to be slightly higher than MIP, similar to recent reports [16]. Kaminska et al. have shown SNIP_O_ to be slightly lower than MIP [10], leading us to conclude that both variables maybe very similar in magnitude. Therefore, SNIP_O_ may help to determine whether a low MIP is due to inspiratory muscle weakness or issues such as difficulty performing MIP. SNIP_O_ may therefore be valuable as part of a multimodal evaluation during diagnosis and monitoring of conditions including NMDs [17].

Despite reduced risk of RMS underestimation, one limitation when using SNIP_O_ is the method of occlusion. During our study, subjects covered the nostril entrance with their thumb. We had decided not to use a plug, as we were concerned this might interfere with the SNIP probe. Tape was also decided against as it allowed leaks and would have been uncomfortable for subjects. In our cohort of healthy subjects, we felt using their thumbs was the most convenient option, however we do understand that this might not be feasible in a patient population with NMD. However, using the thumb method may have decreased reproducibility due to subjects potentially moving their thumb between repeats, reducing certainty that a complete seal was always formed. Although the method of occlusion is not always reported, we noted that Tilanus et al. used a silicone plug for contralateral nostril occlusion [8]. For SNIP_O_ to be performed reliably, a standardized method of occlusion is needed which is compatible with the SNIP probe in use.

### Comparison of SNIP_L_ and SNIP_R_

SNIP_L_ and SNIP_R_ did not significantly differ, suggesting that use of either nostril is appropriate. Other groups have used the nostril appearing most patent [4, 10, 17], however we suggest that giving subjects the choice of nostril for SNIP is a suitable approach. This may be beneficial as some subjects commented that one nostril felt more uncomfortable than the other. Feedback from our subjects suggests that if subjects perform SNIP in the nostril which feels most comfortable, they are more likely to apply maximal effort, increasing SNIP accuracy and hence reducing risk of RMS underestimation. Having a choice would have been particularly useful for two of our subjects who had reported a previous broken nose. Both found difficulty inserting the probe into one nostril, potentially due to previous damage to their nasal structure [29]. In these two cases, the subjects became frustrated and needed more time for nasal probe positioning. To use only the other nostril would have prevented this difficulty, increasing ease of performance and likelihood of maximal effort being applied. Dominant nostril airflow is unlikely to have affected our data, as nostril airflow has been thought to alternate over a short timespan [30].

### Impact of a learning effect or fatigue on SNIP

Within our study, there was a learning effect within the first set of twenty SNIPs. We suggest that 20 repeats are necessary for reliable SNIP testing, in slight contrast with Lofaso et al. who recommend that 20 repeats are necessary only when SNIP_max_ in the first 10 is slightly below normal [19].

SNIP did not decline during 80 repeats, suggesting that healthy subjects are unlikely to experience fatigue during repeat SNIP testing. This confirms and expands upon Uldry and Fitting’s findings that SNIP did not decline during 30 repeats [9]. However, both Uldry and Fitting’s study [9] and the present study included only healthy subjects; we therefore cannot conclude whether fatigue would occur during large numbers of repeats in fatigue-prone subjects, such as NMD patients. Further studies evaluating the impact of fatigue on SNIP in such patients would be beneficial, to ensure that a suitable number of repeats is performed in clinical settings and reliable results are obtained.

### Variation of SNIP depending on gender, ethnicity and age

In our study, SNIP did not differ depending on gender, in agreement with previous findings [10, 20]. However, some groups [27, 31] have reported that SNIP is greater in males. This disagreement is likely due to methodological variation and potentially differing subject demographics. Additionally, we found that SNIP did not correlate with age; supporting previous findings [10, 20]. However, two studies reported a negative correlation with age [9, 27]. These latter two studies [9, 27] did use subjects up to the age of 80, while the former two [10, 20] and our study tended to have subjects or patients younger than 65 years old. This could explain this discrepancy with regard to age.

### Comparison of SNIP and MIP

It is widely reported that SNIP is an easier, more natural test than MIP, which is particularly beneficial when assessing certain subject groups, such as those with NMDs [4]. Previous studies have found such subjects are almost always able to perform SNIP adequately, and always more frequently than MIP [15]. In contrast, all subjects in the present study successfully performed MIP, whereas one subject was unable to successfully perform SNIP. However, the subjects participating in our study were healthy, therefore would not be expected to experience significant difficulty with MIP performance. It is unclear why one subject in our study experienced difficulty with SNIP performance; further studies may help to determine why SNIP may be inappropriate in some subjects or patients and if this could be predicted. Although our study showed SNIP and MIP values were significantly correlated, the Bland–Altman analysis showed they differed, where SNIP was higher. This is supported by previous work, which also found SNIP to be significantly higher than MIP [9]. However, in contrast, work in NMD patients and healthy subjects has shown no significant difference between SNIP and MIP in both groups [10]. Recent work in patients has again shown no significant differences between these two variables [11].

## Limitations

One limitation of our study was that after several SNIP repeats, excess mucus was produced in some subjects, increasing the chance of the probe falling out. This necessitated them to push it back in, often inadvertently changing its position. Different positions might produce different results; we noted if the probe hit the back of the nostril, SNIP was zero as no pressure could be recorded. In contrast, if the probe was not pushed in far enough, it might lower SNIP due to an incomplete seal. Therefore, in order to ensure SNIP remains reliable no matter how many repeats are performed, a technique to ensure the probe remains in a constant position should be devised. Another potential solution is to move the probe to the other nostril if excess mucus is produced.

Another limitation is that, although all subjects performed SNIP whilst in an upright position, their exact position was not strictly controlled. Recent work has shown that a slouched sitting position can significantly decrease SNIP compared to an upright sitting position [22], potentially due to reduced diaphragm tension and movement. Thus, variations in subject position may have introduced variability into our results and affected the reliability of our study.

Only healthy subjects were included in the present study. Results would differ significantly in patients with diseases such as NMDs, for example due to muscular weakness and increased likelihood of fatigue, among other reasons [12]. Although our conclusions cannot be extended to patient populations, they do provide a baseline to guide the collection of normal values in healthy subjects, and this in turn will aid diagnosis and prognosis in patients with reduced SNIP values.

Inter-investigator reliability was not an issue, as our study only had one investigator involved in data collection.

## Conclusions

In summary, we showed that SNIP testing via either nostril, with the contralateral nostril occluded, is an appropriate and reliable method of assessing respiratory muscle strength and that SNIP values are higher to MIP values. We also conclude that twenty repeats are sufficient to overcome any learning effect and that fatigue is not an issue in healthy subjects even during up to 80 repeats. Our subset study confirmed that SNIP is associated with minimal fatigue as the shortest rest interval between SNIP repeats (30 s) did not affect results, suggesting SNIP performance does not require significant effort in healthy subjects. A larger cohort of patients would be required to ideally optimize measurements of SNIP measurement methodology. We believe our findings are important for methodological studies determining reference values in a healthy population (Additional file 1).

## Supplementary Information

**Additional file 1.** **Supplementary data file 1.** This file show all the data for the 51 subjects who were selected in this study.

## Data Availability

The data that support the findings of this study are available from the corresponding author upon reasonable request.
